# An Enhanced Platform to Analyse Low-Affinity Amyloid β Protein by Integration of Electrical Detection and Preconcentrator

**DOI:** 10.1038/s41598-017-14338-4

**Published:** 2017-10-30

**Authors:** Yong Kyoung Yoo, Dae Sung Yoon, Gangeun Kim, Jinsik Kim, Sung Il Han, Junwoo Lee, Myung-Sic Chae, Sang-Myung Lee, Kyu Hyoung Lee, Kyo Seon Hwang, Jeong Hoon Lee

**Affiliations:** 1 Department of Electrical Engineering, Kwangwoon University, 447-1 Wolgye, Nowon, Seoul, 01897 South Korea; 2 Department of Clinical Pharmacology and Therapeutics, College of Medicine, Kyung Hee University, Seoul, 02447 South Korea; 3 School of Biomedical Engineering, Korea University, Seoul, 02841 South Korea; 4 Department of Medical Biotechnology, College of Life Science and Biotechnology, Dongguk University, 10326 Seoul, South Korea; 5 Department of Chemical Engineering, Kangwon National University, Gangwon-do, 24341 South Korea; 6 Department of Nano Applied Engineering, Kangwon National University, Gangwon-do, 24341 South Korea

## Abstract

Sensitivity and limit of detection (LOD) enhancement are essential criteria for the development of ultrasensitive molecular sensors. Although various sensor types have been investigated to enhance sensitivity and LOD, analyte detection and its quantification are still challenging, particularly for protein-protein interactions with low association constants. To solve this problem, here, we used ion concentration polarization (ICP)-based preconcentration to increase the local concentration of analytes in a microfluidic platform for LOD improvement. This was the first demonstration of a microfluidic device with an integrated ICP preconcentrator and interdigitated microelectrode (IME) sensor to detect small changes in surface binding between antigens and antibodies. We detected the amyloid beta (Aβ) protein, an Alzheimer’s disease marker, with low binding affinity to its antibodies by adopting ICP preconcentration phenomena. We demonstrated that a combination of ICP preconcentrator and IME sensor increased the LOD by 13.8-fold to femtomolar level (8.15 fM), which corresponds to a significant advance for clinical applications.

## Introduction

Biological interactions between target analytes related to lethal diseases and their antibodies have attracted much attention of numerous researchers, and are critical for detection of many biomarkers and diagnosis of clinically important diseases^1–3^. Biomarker detection, which mainly indicates protein–protein interaction events, holds great promise for the detection of diseases or physiological dysfunction^4–8^. However, the fact that numerous antibodies have a wide range of binding affinity to their target antigens due to their own immunological origins, may act as a major hurdle in clinical area. Because early diagnosis and personalized treatment are often based on multiplexed detection of tens or hundreds of biomarkers^9–11^, this problem become more significant (especially in very low affinity range). For example, in Alzheimer’s disease (AD), biomarker-based detection and treatment in presymptomatic (i.e. predementia) stages might be most effective^9,12^, before amyloid plaques become widespread. Amyloid beta (Aβ) is an AD biomarker^13,14^; its antibody has a high dissociation constant (K_D_ = ~22.3 nM; 100.68 ng/mL), suggesting low-binding-affinity interactions^15^. Currently, the clinically relevant range of Aβ is several several ten to several hundred pg/mL, which is beyond the analytical sensitivity for detection supported by current detection methods.

Though many research groups have investigated the detection of biomarkers at low concentrations^15–17^, poor protein-protein interactions due to low binding affinities and high dissociation constants are still a great hurdle for molecular detection methods. Biotin-streptavidin interaction is known to be a typical example for high binding affinities and extremely low dissociation constants (K_D_ = ~250 fM)^18^. Below the typical range (10^−8^ to 10^−12^ M) of dissociation constants (K_D_), molecular interactions between analytes and their receptor materials (mainly, antibodies) become minimal, leading to weak binding signals^19^. Many researchers reported that nano-scaled sensors, e.g. silicon nanowires and carbon nanotubes^20–24^, have improved limits of detection (LOD) in the case of low affinities. However, these sensors have several limitations, including a narrow dynamic range, owing to their small surface areas. Alternatively, Ab engineering may facilitate low-concentration sensing, but is not always feasible.

Sample pre-treatment with ion concentration polarization (ICP)-based preconcentration at binding stages^25–29^ can be considered as one of the methods to overcome low binding affinities^19,30,31^. However, these studies have focused mainly on fluorescently labelled preconcentrators that are not suitable for label-free electrical detection. Here, we speculate that an integrative device with electrical sensors for preconcentration and the detection of low-affinity targets, can solve the above mentioned hurdle, improving the LOD and reliability. Label-free biosensors have various benefits; they are low-cost point-of-care diagnostic devices with high sensitivity. Therefore, we used interdigitated microelectrodes (IME) as an electrical sensing platform^32^. A two-stage approach provided a locally analyte-concentrated area for sensing, and the label-free biosensors detect the highly concentrated analytes after biomarker preconcentration. If the above concept is realized, this correspond to the first practical demonstration of ICP preconcentrator-based electrical detection.

For validation of our suggested concept, we fabricated IME-embedded microfluidic chips with individual preconcentrator elements that were electrically driven by ICP. The AD causing material, Aβ was used as a model biomarker, which has an extremely low association constant. We investigated how much the LOD is improved by adopting the preconcentrator^33^, and whether a combination of the IME microfluidic chip and the preconcentrator is really compatible and makes the assay process simple. To analyse the effect of locally increased concentrations, we discuss the experimental results in terms of two crucial aspects: the quasi-macromolecular crowding effect and free energy^34^. In this paper, we demonstrate that use of the preconcentrator significantly improves the LOD and sensitivity of the IME microfluidic chips (by 13.8-fold to femtomolar levels) and makes the assay process more reliable, effectively lowering coefficient of variance. To the best of our knowledge, this is the first reported use of a label-free preconcentrator integrated sensor to overcome the LOD for extremely low association constants.

## Results and Discussion

**Figure 1:**
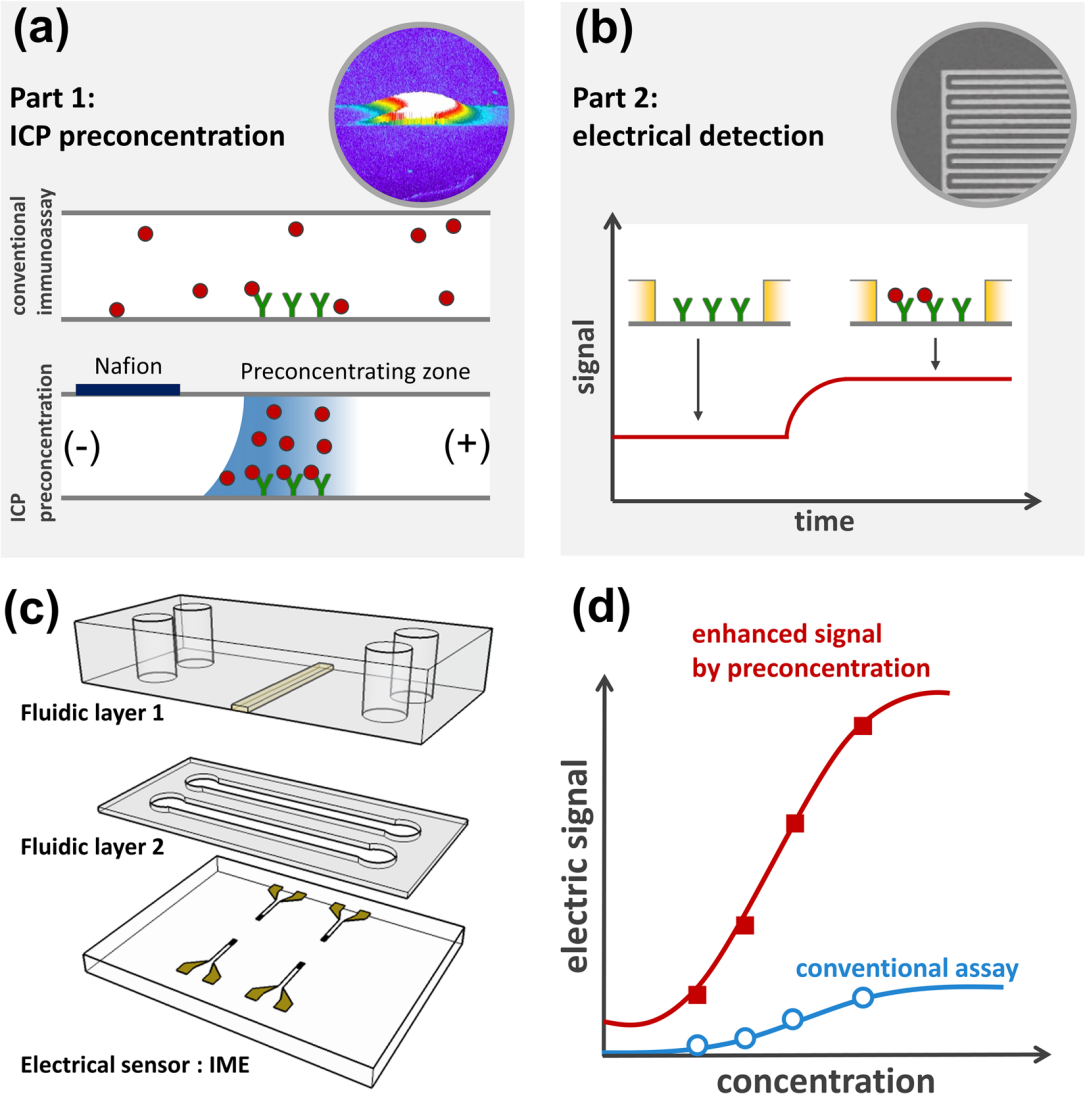
The ion concentration polarization (ICP) preconcentrator-integrated sensor system with interdigitated microelectrode (IME) enabled the detection of Aβ. (**a**) Preconcentrator, molecule preconcentration scheme, and molecular sensing; (**b**) biomolecular sensor using the IME sensor (electrical sensor); (**c**) ICP preconcentrator-embedded IME sensor. Fluidic layer 1 (sample reservoir and ion-permselective materials for ICP), fluidic layer 2 (sample delivery to the IME and ICP), and electrical sensor for Aβ detection; (**d**) performance enhancement utilizing the ICP preconcentrator-embedded IME sensor.

To confirm whether our suggested concept is relevant, we proceeded to fabricate an ICP preconcentrator-integrated IME sensor built in the microfluidic device (Fig. 1). The sensor consisted of two functional parts, the ICP preconcentrator for Aβ collection (Fig. 1(a)) and the electrical biosensor for Aβ detection (Fig. 1(b)). The ICP preconcentrator had a PDMS microfluidic channel (Fluidic layer 2: Sample flow channel in Fig. 1(c)) and an ICP-inducing layer composed of ion-permselective materials (Fluidic layer 1: ICP-inducing layer in Fig. 1(c)) on a sensor surface. ICP preconcentration phenomena taking place on a sensing zone are expected to increase assay sensitivity and the LOD as shown Fig. 1(d).

**Figure 2:**
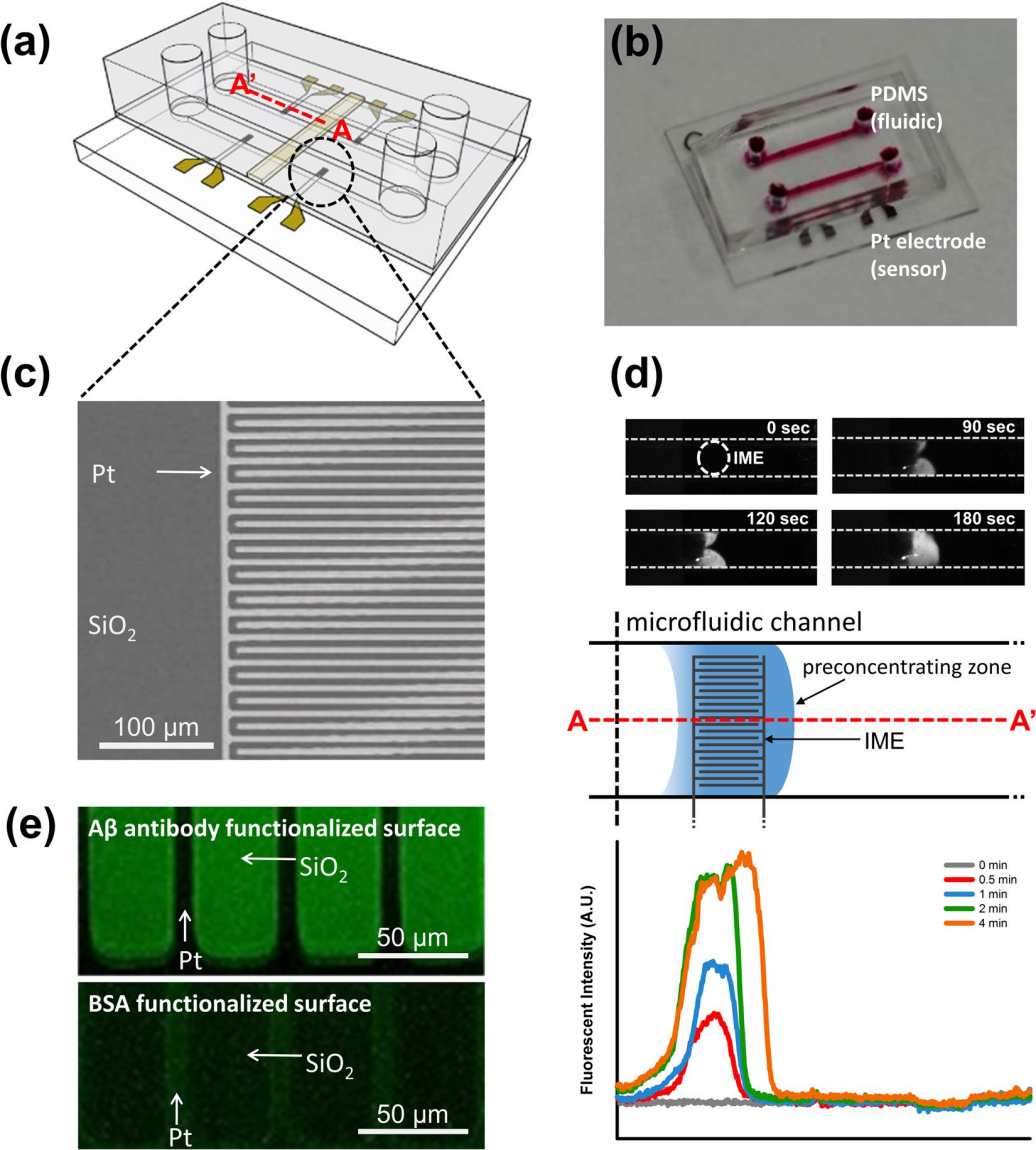
The ICP preconcentrator-integrated sensor system. (**a**) The system was composed of 4 IMEs on a chip and 2 microfluidic channels for the ICP preconcentration phenomena. In a microfluidic channel, one IME was utilized for the antibody against the binding protein and the other IME was utilized for the reference electrode. (**b**) Image of the ICP preconcentrator-integrated sensor system with IME. (**c**) Scanning electron microscope image of IME. (**d**) Fluorescence images of Aβ preconcentration on IME, scheme of the ICP preconcentration phenomena on IME, fluorescent intensity of Aβ on IME and the location of the preconcentration plug over time. (**e**) Aβ antibody functionalization and BSA binding to block nonspecific binding.

The width and depth of the microchannel were approximately 650 μm and 100 μm, respectively (Fig. 2(a)). An electrical field was applied for ICP at the ends of reservoirs using silver chloride (Ag/AgCl) electrodes. We induced a large volume of preconcentration plug in the preconcentrator using pressure-driven flow (1.3 μL/min) and measured Aβ and Aβ antibody interactions. Pressure was applied using a syringe pump. Based on a previous report, it has been known that protein-protein interactions on an electrode can increase impedance^35^. To avoid the mentioned problem, instead, we immobilized the Aβ antibody on a silicon dioxide (SiO_2_) surface between electrodes, as shown Fig. 1(e). We confirmed the immobilization of the Aβ antibody on the SiO_2_ surface. We obtained fluorescent images (Fig. 2(e)) for two surfaces, i.e. an Aβ antibody surface and a bovine serum albumin (BSA)-functionalized surface (see Supplementary Information). The Aβ antibody-functionalized surface was verified using the fluorescein isothiocyanate (FITC)-tagged Aβ reaction. The low fluorescein intensity of the BSA-functionalized surface as a negative control was measured using the FITC-tagged Aβ reaction. For label-free electrical detection of preconcentrated Aβ, we fabricated an IME sensor with a multi-layered thin film, Si/SiO_2_/Ti/Pt (500 μm/300 nm/30 nm/150 nm), as shown in Fig. 2(c). We optimized the dimensions of the finger electrode to 300 × 5 μm (length × width). The gap between electrodes was 5 μm and the number of fingers was 60. The Aβ antibody was immobilized on the SiO_2_ surface between finger electrodes via aldehyde groups. The detailed protocol and optimized design are described elsewhere^33^. The IME-patterned glass was bonded with two-layered PDMS using a plasma bonder and the final device is depicted in Fig. 2(b). We observed the control signal for interactions between Aβ samples and anti-prostate specific antigen (PSA) in the top PDMS channel, and we used Aβ samples to measure Aβ antibody signals in the bottom channel. We calculated signal differences as a net signal between control and Aβ samples, thereby eliminating the effects of signal drift, noise, and non-specific information.

To solve assay problem due to low binding affinity, we performed Aβ preconcentration using ICP. The exact location of the preconcentration plug on the IME sensor is important for device performance; accordingly, we optimized the combination of applied field/pressure-driven flow, so that the optimal conditions were adjusted to 200 V/cm and 1.3 μL/min, respectively. Preconcentration increased the Aβ (pI = 5.31, Mw = 4514.64 Da) concentration from approximately 155 pM to 3.87 nM in the IME sensing zone, resulting in Ab–analyte conjugation. We observed an Aβ increase of at least 25-fold (see Supplementary Information). The Aβ preconcentration plug formed on the IME sensor spanned 1 mm in length, and stably located in the sensing zone, as shown Fig. 2(d). We optimized preconcentrating stability by applying pressure driven flow (See S.I. Fig. S6). This Aβ preconcentrated plug could enhance the low binding affinity, and therefore pushed the detection limit toward the clinically meaningful range of several dozen to several hundred pg/mL for AD.

We showed fluorescence image that confirm Aβ antibody immobilization (Fig. 2(e)). Top image was taken after FITC labelled Aβ interaction with immobilized Aβ antibody. Bottom images are from FITC labelled Aβ interaction with immobilized BSA. We observed clear differences that confirm surface immobilization. We analysed XPS spectra of the functionalized surface that revealed that C and N peaks in the wide range, while no or very small peak was founded in bare SiO_2_ surfaces (See S.I. Fig. S6(a)).

**Figure 3:**
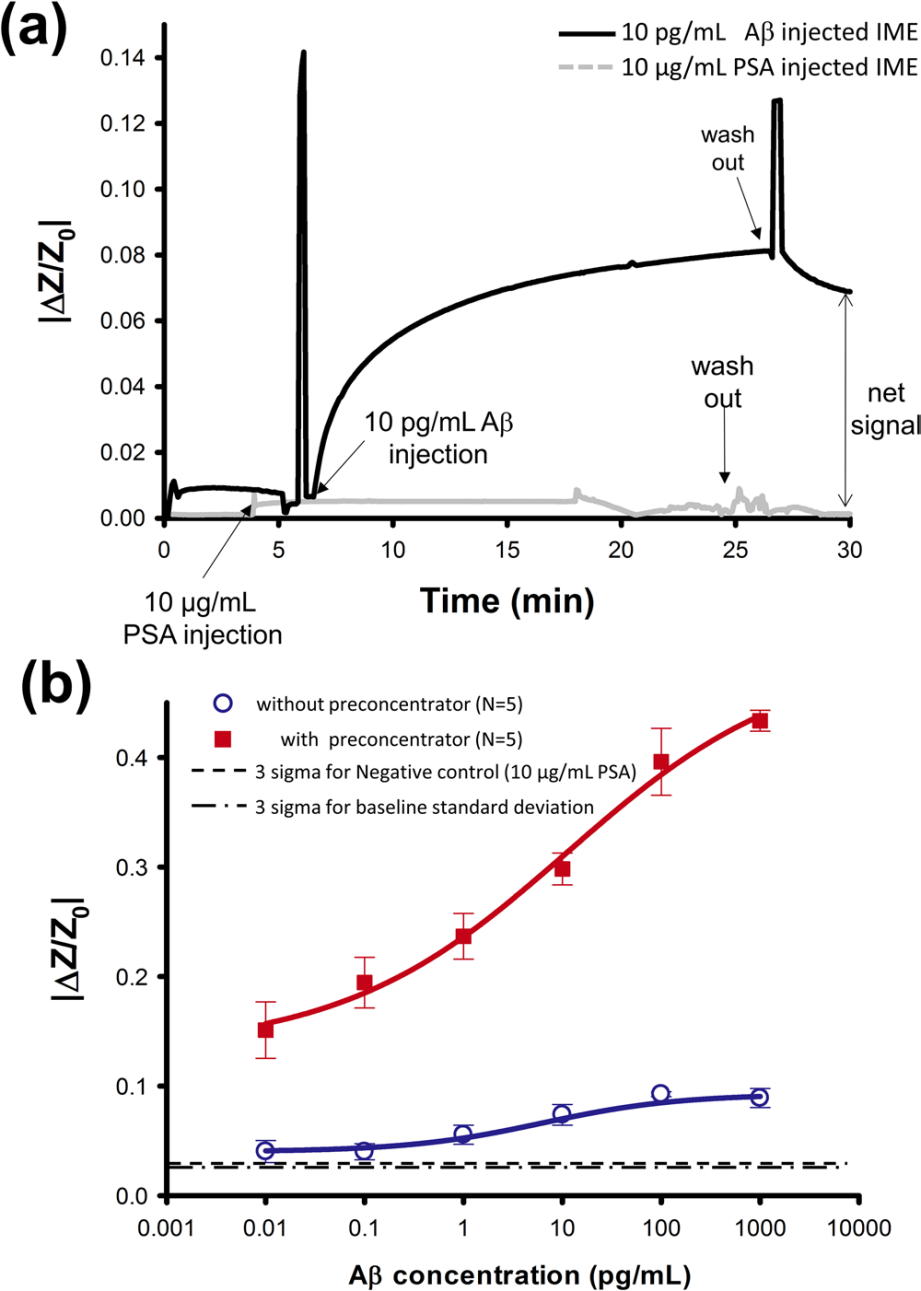
System performance with respect to Aβ detection. (**a**) Real-time detection of 10 pg/mL Aβ and 10 μg/mL PSA. The net signal differential was calculated between the sample and control. (**b**) Electrical signal versus Aβ concentration using a preconcentration step (red; squared), showing great increase in sensitivity and LOD while no significant increase in slope without a preconcentration step (Blue; open dot). Three sigma based lines of standard deviation were calculated both from mean value of 0.1 pg/mL Aβ signal. Three sigma for negative control (10 μg/mL PSA) were calculated with the standard deviation.

To check the interactions between Aβ Ab and Aβ (selectivity), we monitored electrical signals with both 10 pg/mL Aβ and 10 μg/mL PSA. For comparison with the ICP cases, all the experiments here were carried out with IME only (without ICP components). We prepared the sensor surface with the same Aβ Ab receptor, which showed typical IME signals from real time monitoring (Fig. 3(a)). We observed that the increased signal was measured at 10 pg/mL Aβ whereas no signal increase at 10 μg/mL PSA, implying that the Aβ Ab receptor retains good selectivity.

With ICP operation, the background noise in the electrical signal could increase because increasing background concentrations of compounds (including analytes) produced additional electrical noise. Moreover, the applied DC field from ICP preconcentration affected the commercial AC measuring system in the IME sensors. We therefore conducted all assays with net signals (see Fig. 3(a)) and show electrical signals from Aβ concentration in Fig. 3(b). Based on a commercially accepted protocol such as ELISA, we could evaluate the sensitivity and LOD of the ICP preconcentrator-embedded IME sensor for target Aβ with low binding affinity. We measured net signals during 5 min of preconcentration, and Aβ concentrations ranged from 10 fg/mL to 1 ng/mL.

Free energy and entropy, especially in protein–protein interactions, are correlated with binding kinetics^36–39^. Assuming that a system is isolated, the entropy term is described by statistical mechanics with the assumption of an equally microscopic probability^40^. Based on the ICP preconcentration results, the entropy of the ICP preconcentration plug increased the complexity and population of molecules. The binding affinity of protein–protein interactions is defined as follows:

1 $$R+L\rightleftharpoons RL$$

2 $${k}_{on}=\frac{[RL]}{[R][L]}={k}_{off}$$

3 $${k}_{d}=\frac{{k}_{off}}{{k}_{on}}=\frac{[R][L]}{[RL]}$$

where *k*_*on*_ is the association rate constant, *k*_*off*_ is the dissociation rate constant, and *k*_*d*_ is the equilibrium binding constant. The binding affinity of protein–protein interactions is affected by the equilibrium binding constant of free energy^37,40^ as follows:

4 $${\Delta }G=RT\,\mathrm{ln}\,\frac{{k}_{d}}{{c}_{0}}=\Delta H-T\Delta S$$

5 $${k}_{d}=\frac{e({\Delta }H-T{\Delta }S)/RT)}{{c}_{0}},$$

where *∆G* is free energy, *R* is the gas constant, *T* is temperature, c_0_ is 1 mol/L, and *∆H* is Enthalpy, in Equations 4 and 5. According to Equation (5), under constant temperature, the ICP preconcentration phenomenon is expected to increase entropy, leading to an exponential decrease in *k*_*d*_. As with the macromolecular crowding effect, the ICP preconcentration enhances reaction rates owing to a reduction in the total excluded volume, i.e. the volume that is inaccessible to other molecules in the system as a result of the presence of the first molecule^41^ and we defined it as a quasi-macromolecular crowding effect. Macromolecular crowding effect is defined as changes in molecular properties such as the rates and equilibria of interactions in a solution with high concentrations of macromolecules (see Fig. 1(d)). Such phenomena have been widely accepted in biochemistry and cell biology. Based on Equation (3), a decrease in *k*_*d*_ increases binding efficiency of low affinity molecules, which enhances both the sensitivity and LOD of immunoassays, and the assay time can be shortened by monitoring impedance signals. The ICP-based integrated IME sensor could provide an electrical signal, resolving limitations concerning the target concentration and association rate between analytes and recognition material. Moreover, we realized a simple and efficient method of integration between the ICP preconcentrator and IME sensor platform, without requiring optical components.

We measured the absolute values of ∆Z/Z_0_ (|∆Z/Z_0_|) using the IME sensors (Fig. 3(b); presented as averages for 5 devices) with and without preconcentration steps. Impedance without preconcentration revealed 4.0 ± 1.0%, 4.0 ± 0.7%, 5.5 ± 0.9%, 7.3 ± 0.9%, 9.2 ± 0.3%, and 8.9 ± 0.9% increases for 10 fg/mL, 100 fg/mL, 1 pg/mL, 10 pg/mL, 100 pg/mL, and 1 ng/mL Aβ, respectively. The relative standard deviation (RSD) without preconcentration was calculated as 50.1, 36.7, 31.2, 25.6, 4.7, and 19.4% for 10 fg/mL, 100 fg/mL, 1 pg/mL, 10 pg/mL, 100 pg/mL, and 1 ng/mL Aβ, respectively. The regression equation were calculated to |∆Z/Z_0_| = (0.001471 ± 0.00212) * log(Aβ concentration) + (0.06141 ± 0.00408). The slope of the linear regression of Aβ concentration on impedance change in the linear range of 1 pg/mL to 1 ng/mL was 0.0147 (Fig. 3(b); see Supplementary Information). Correlation coefficient (Pearson’s r) and coefficient of determination (R^2^) were estimated to 0.9610 and 0.9044. The dynamic range measured without preconcentration was 3 orders of magnitude (in the linear range from 1 pg/mL to 100 pg/mL); however, resolution was insufficient for discrimination below clinical relevant concentrations of 100 fg/mL Aβ. To define the LOD, we used two control experiments. First, we measured the interaction between the Aβ antibody and highly concentrated (i.e. 10 μg/mL) PSA protein as a negative control. The LOD, as shown in Fig. 3(b), was defined as three sigma for the signal to noise ratio based on the control^42^. From the crosspoint of 100 fg/mL and 3 sigma for impedance change. Impedance with preconcentration, which leads to ICP preconcentration phenomena, is shown in Fig. 3(b). The |∆Z/Z_0_| values were 15.1 ± 2.5%, 22.9 ± 2.3%, 23.0 ± 2.0%, 30.1 ± 1.4%, 39.6 ± 3.0%, and 42.8 ± 0.9% for 10 fg/mL, 100 fg/mL, 1 pg/mL, 10 pg/mL, 100 pg/mL, and 1 ng/mL Aβ, respectively. The RSD with preconcentration was down to 33.8, 23.8, 17.6, 9.7, 15.3, and 4.3% for 10 fg/mL, 100 fg/mL, 1 pg/mL, 10 pg/mL, 100 pg/mL, and 1 ng/mL Aβ, respectively, indicating a more reliable sensor performance. The regression equation were calculated to |∆Z/Z_0_| = (0.06018 ± 0.00325) * log(Aβ concentration) + (0.25072 ± 0.0074). Sensitivity was defined as the linear slope of the regression of signal on concentration (see Supplementary Information). Pearson’s r and R^2^ were estimated to 0.9942 and 0.9855. Based on the logarithmically linear slope of 0.0601 for the range of Aβ concentrations, we expect an increase in sensitivity of up to 4.09-fold. In addition, we observed a 13.8-fold increase in the LOD down to 36.8 fg/mL (See Supplementary Information for detailed LOD evaluation). Moreover, extrapolating from the regression, we expected increases in the LOD to several hundred attomolar.

## Conclusion

In summary, we investigated an enhanced immunoassay platform, with particular applications to molecules with low dissociation constants, utilizing ICP preconcentration. Utilizing the ICP preconcentration phenomena, on binding affinity, we showed 4.09- to 13.8-fold increases in the LOD. Owing to the enhanced LOD and dynamic range, the new device platform is useful for clinically meaningful concentrations. The ICP preconcentrator with electrical detection provides a valuable platform for biomolecule detection, especially for body fluids with non-abundant target biomolecules, such as saliva, cerebrospinal fluid, and urine.

## Methods

### IME chip and ICP preconcentrator fabrication

The IME sensor layer was fabricated following a standard microfabrication process. A glass wafer substrate was used, enabling ICP preconcentration monitoring using an inverted fluorescent microscope. For the electrode materials, platinum (Pt, 150 nm) and titanium (Ti, 30 nm) were deposited on the glass using a sputtering process. The Pt/Ti layer was patterned using photolithography (MA6; Karl Suss, Munich, Germany) and etched using an inductively coupled plasma-reactive ion etcher (Oxford Instruments, Abingdon, UK). The IME chip was composed of 4 IME pairs of 5 μm in width and 300 μm in length, with a 5-μm gap between electrodes. The design was optimized with 30 finger pairs (published separately). The ICP preconcentrator was composed of 2 PDMS layers. Fluidic layer 1 contained an embedded ion-permselective membrane (Nafion perfluorinated membrane, thickness: 180 μm; Sigma-Aldrich, St. Louis, MO, USA) and reservoirs for ICP operation. Fluidic layer 2 was designed for flowing biomolecule sample delivery (see Supplementary Information). The layers were bonded using a plasma bonder (CUTE; Femto Science Inc., Somerset, NJ, USA) and the integrated fluidic layer was bonded with precut double-sided tape (Scotch, 3 M). An electronic cutting machine (CAMEO, Silhouette Inc., Lehi, UT, USA) was used to cut pieces of 650 μm in width, 950 μm in length, and 90 μm in thickness. 3 M tape was used instead of plasma bonding, since the IME surface contained immobilized Aβ antibody.

### Functionalization of IMEs

To form hydroxyl groups, we first cleaned IME-patterned glass wafers using piranha cleaning solution (5:1 ratio of sulfuric acid and hydrogen peroxide) for 30 min, as shown Fig. S1(a). To form amine functional groups on the SiO_2_ surface, the cleaned surface was dipped in 3-(ethoxydimethylsilyl)propylamine solution (APMES; 1% in isopropyl alcohol (IPA); Sigma-Aldrich) for 3 h. The IME sensor was chemically modified using polyvinyl pyrrolidone-aldehyde solution (PVP-CHO; 10 mM in 100 mM NaHCO_3_ solution; pH 9.0) for 6 h, followed by sodium borohydride solution (NaBH_4_, 10 mM in 100 mM NaHCO_3_) for 1 h. For linker formation, IME was treated with 1% glutaraldehyde solution in 100 mM NaHCO_3_. Finally, the Aβ antibody (6E10; monoclonal antibodies for specific binding to the first 16-peptide-residue of amyloid β peptides, Covance) was immobilized on the aldehyde group on SiO_2_ for specific binding to the Aβ protein fragment 1–42. Bovine serum albumin (BSA, Sigma-Aldrich) was used to block non-specific binding. The Aβ protein was prepared in a 0.1 × PBS buffer solution at concentrations ranging from 10 fg/mL to 1 ng/mL. The graph of fluorescence intensity against selectivity (Fig. 2(e)) was used to quantitatively analyze Aβ binding, as shown Fig. S1(b).

### Preconcentration and Aβ Assay

To perform Aβ assays of IME sensors with and without preconcentration-enhancement, we first measured the impedance of IMEs in a 0.1 × PBS buffer solution before Ag-Ab interactions, and then determined Ag-Ab interaction via Aβ sample injection. After washing out the sensor surface with 0.1 × PBS buffer solution, we measured two electrical signals (before and after Ag-Ab interactions) and used the difference as assay signal (see net signal indicated in Fig. 3(a)). Because the interference between the dc signal from ICP preconcentration and the ac sensing signal from the IME electrode is critical for sensor output, we could effectively prevent signal interference using the assay protocols described above.

## Electronic supplementary material

Supplementary information
