# Supplementary material for: An Enhanced Platform to Analyse Low-Affinity Amyloid β Protein by Integration of Electrical Detection and Preconcentrator
Source: Sci Rep. 2017 Oct 30;7:14303. doi: 10.1038/s41598-017-14338-4 (PMC5662786; doi:10.1038/s41598-017-14338-4)
Supplement: Supplementary file 1 — Supplementary information [file 41598_2017_14338_MOESM1_ESM.pdf]

An Enhanced platform to Analyze Low Affinity Amyloid β Protein by Integration of Electrical Detection and Preconcentrator

Yong Kyoung Yoo, Dae Sung Yoon, Gangeun Kim, Jinsik Kim,

Sung Il Han, Junwoo Lee, Myung-Sic Chae, Sang-Myung Lee,

Kyu Hyoung Lee, Kyo Seon Hwang, Jeong Hoon Lee

Functionalization of interdigitated microelectrodes

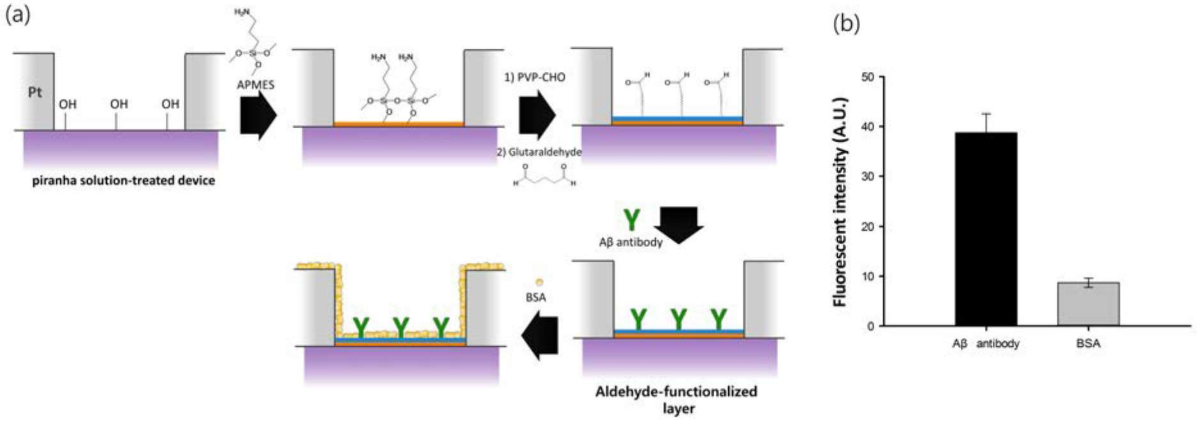

Figure S1. (a) Schematic depiction of IME functionalization. (b) Fluorescence intensity of Aβ antibody functionalization and BSA binding to block nonspecific.

Measurement system

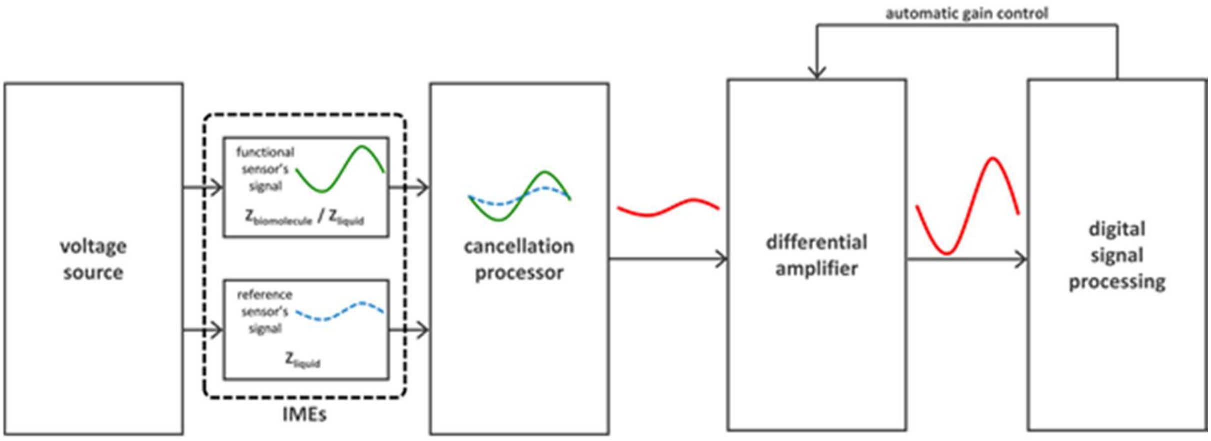

Figure S2. Schematic diagram of the IME impedance measurement system.

Interactions between the Aβ antibody and Aβ were measured using the impedance measurement system for IME sensing. The system was designed with a signal cancellation and amplification system to block noise from parasitic capacitance and measure low-level signals. The noise was attributed to the biomolecule-containing solution and relatively long IME length. In Figure S2, $Z_{biomolecule}$ and Z_{liquid} represent the impedance of the biomolecule and solution, respectively. $Z_{biomolecule}$ and Z_{liquid} are dominant (approximately 1000–2000-fold greater) compared with other impedance components. We developed a cancellation and amplification embedded measurement system to remove Z_{liquid} and other impedance components. The functional electrode, which could detect the impedance of biomolecules and liquids, and the reference electrode, which could detect only the impedance of liquids, were prepared in solution. In the same conditions, after measuring the two impedimetric signals using the functional and reference electrodes, the two signals were subtracted to obtain the changes that were due to biomolecules. The amplification process was applied after cancellation.

The linear relation of sensitivity for Aβ detection

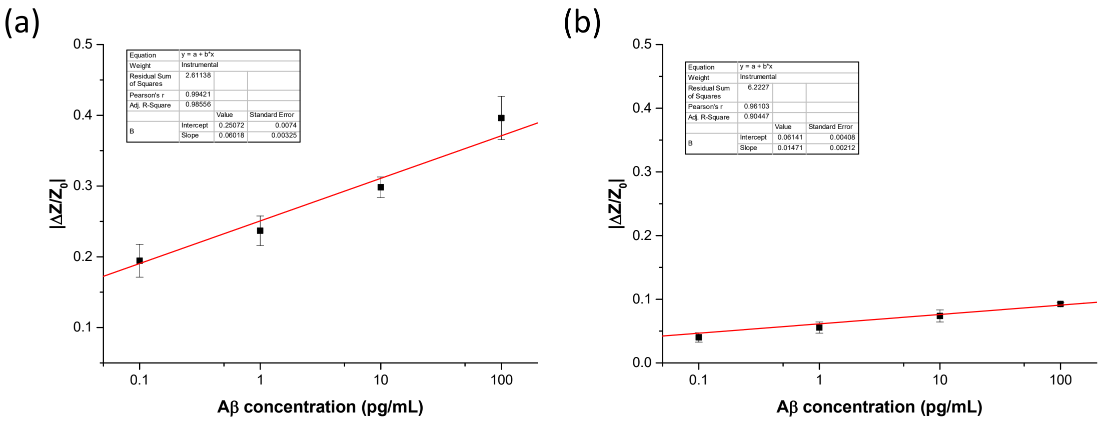

Figure S3. The logarithmically linear relationship between sensitivity and Aβ for 0.1 to 100 pg/mL Aβ. (a) The calibration lines were fitted to $|\Delta Z/Z_0|$ = (0.06018 ± 0.00325) * log(Aβ concentration) + (0.25072 ± 0.0074) for IME impedance with ICP preconcentration(R^2 = 0.98556). (b) $|\Delta Z/Z_0|$ = (0.01471 ± 0.00212) * log(Aβ concentration) + (0.06141 ± 0.00408) without ICP preconcentration (R^2 = 0.90447). And 98.55 % and 90.44 % of the total variation in impedance change can be explained by the linear relationship between Aβ concentration and impedance change.

Selectivity for Aβ detection

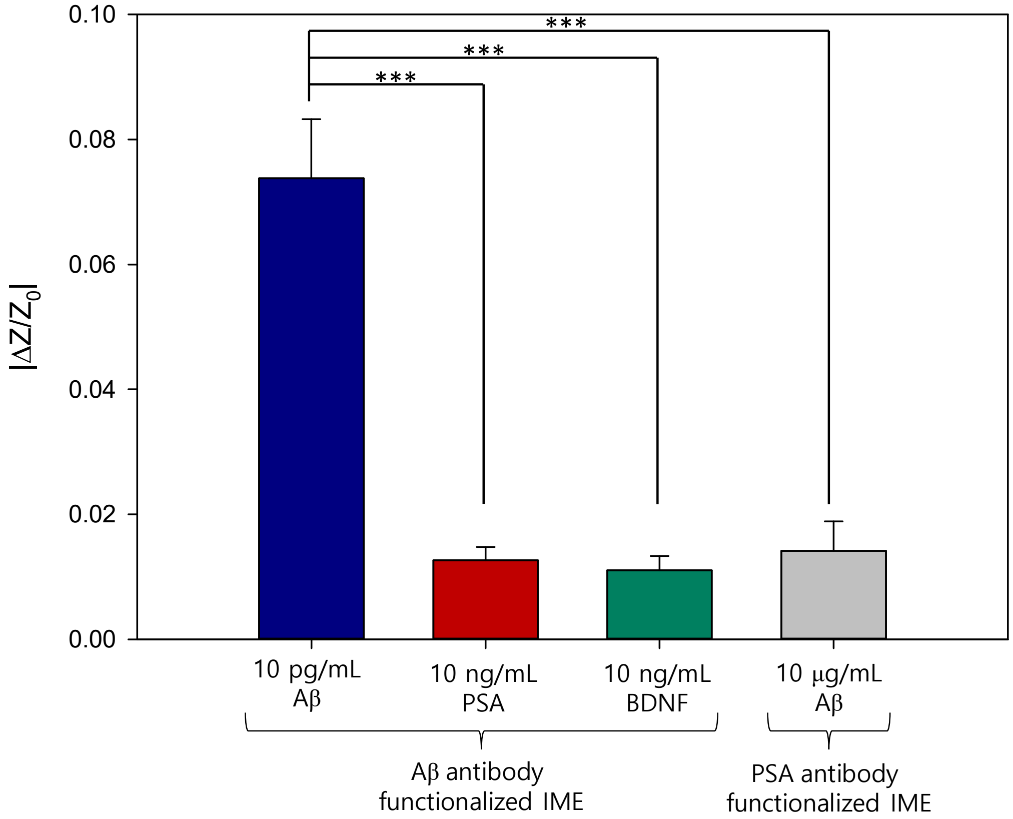

Figure S4. To examine the selectivity of Aβ detection, IME functionalized with the Aβ antibody was prepared. The target sample was injected into the IME sensor without the ICP preconcentrator. Impedance changes of 7.38 ± 0.94%, 1.27 ± 0.21% and 1.11 ± 0.22% respectively, were measured for 10 pg/mL Aβ, 10 ng/mL PSA, and 10 ng/mL brain-derived neurotrophic factor (BDNF) with Aβ antibody functionalized IME. And impedance change of 1.41 ± 0.47% were measured for 10 μg/mL Aβ with PSA functionalized IME. The impedance change from the Aβ reaction was greater than that for other biomolecules (one-way ANOVA). (***, $p < 0.001$, $n = 5$)

Aβ preconcentration

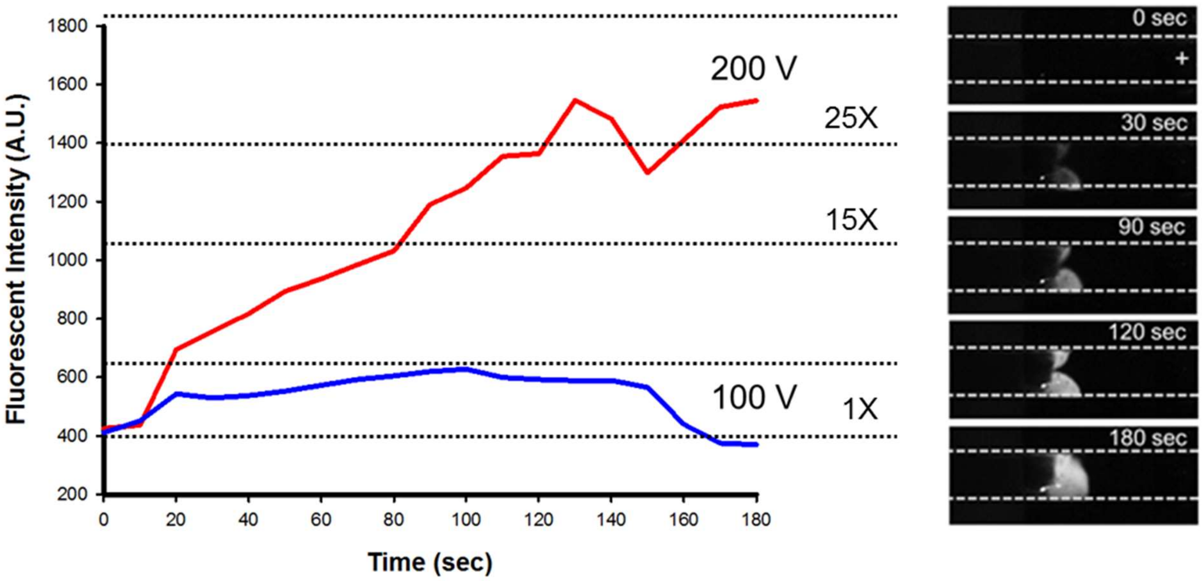

Figure S5. Performance of Aβ ICP preconcentration along with operating voltage for IME. A preconcentration factor of approximately 25-fold was observed at 200 V using a fluorescent microscope. The preconcentration plug was localized on the IME during preconcentration and interactions between the Aβ antibody and Aβ

LOD calculation

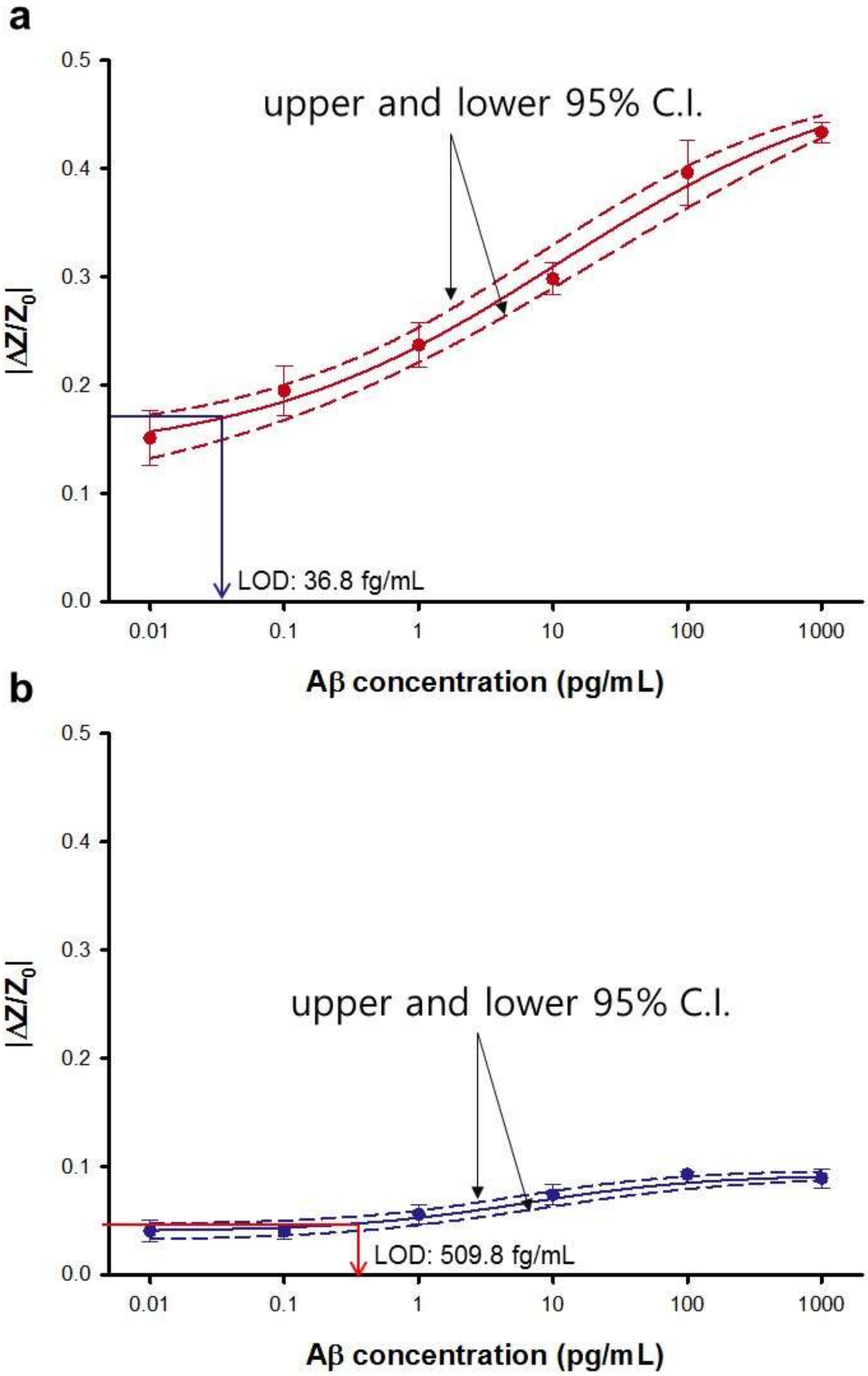

Figure S5. Limit of detection (a) with and (b) without ICP preconcentration. The LOD were evaluated using suggested LOD evaluation method.[1-4] The dashed line of upper and lower 95 % confidence interval (CI) were respectively described. The intersection between upper 95 % CI and standard sensitive curve with and without ICP preconcentration were calculated about 36.8 and 509.8 fg/mL.

XPS spectra of antibody functionalized surface

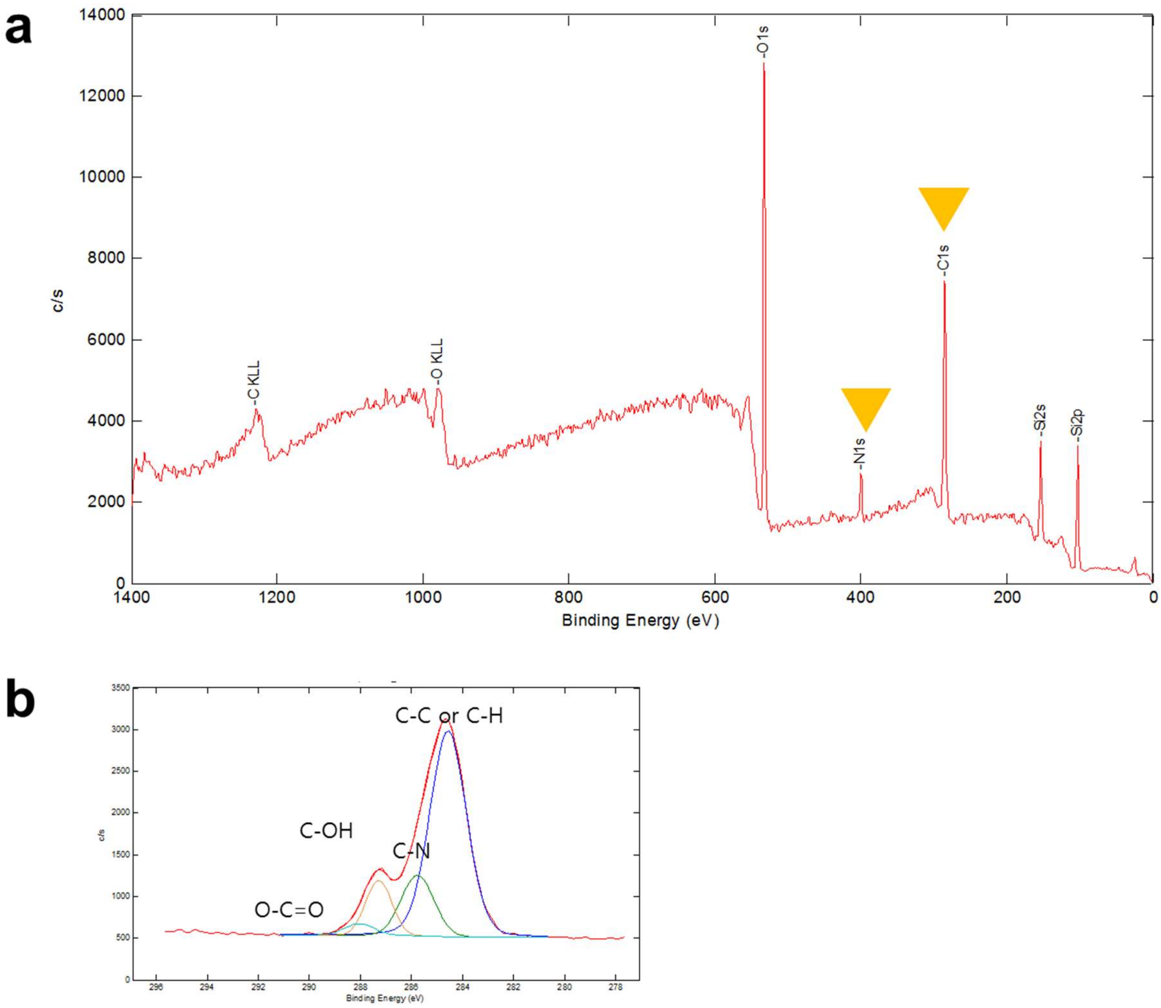

Figure S6. XPS spectra of antibody immobilized SiO_2 surface. (a) XPS peak of antibody immobilized surface. (b) The peak fitting curves of C1s peak.

The XPS spectra of the functionalized surface revealed that C and N peaks in the wide range (Figure S6 (a)), while no or very small peak was founded in bare SiO_2 surfaces. [5-6] Particularly, the narrow spectra of C1s which can be deconvoluted by oxygenated groups and a C–N binding. Moreover, the presence of N1s peak indicates that chemical modifications of SiO_2 surfaces included crosslinking with APTES and glutaraldehyde as well as the existence of immobilized proteins (Figure S6 (b)).

Reference

1 M.A. O'Connell. A. Belanger. P.D. Haaland, Calibration and assay development using the four-parameter logistic model. *Chemometrics and Intelligent Laboratory Systems* **20**, 97 (1993).

2 Conrad P. Quinn *et al.* Specific, Sensitive, and Quantitative Enzyme-Linked Immunosorbent Assay for Human Immunoglobulin G Antibodies to Anthrax Toxin Protective Antigen. *Bioterrorism-related Anthrax* **8**, 1103 (2002).

3 Blake Farrow *et al.* A Chemically Synthesized Capture Agent Enables the Selective, Sensitive, and Robust Electrochemical Detection of Anthrax Protective Antigen. *ACS NANO* **7**, 9452, (2013).

4 G.S. Sivko, *et al*. Evaluation of early immune response-survival relationship in cynomolgus macaques after Anthrax Vaccine Adsorbed vaccination and Bacillus anthracis spore challenge. *Vaccine* **34**, 6518, *(*2016).

5 Ghosh, R., Ezhilvalavan, S., Golding, *et al*. Profiling of the SiO2 - SiC Interface Using X-ray Photoelectron Spectroscopy. *MRS Proceedings*, 640, (2000)

6 Agarwal, Dilip Kumar, *et al*. Asymmetric immobilization of antibodies on a piezo-resistive micro-cantilever surface. *RSC Advances* 6.2, 17606 (2016)
